# A Bayesian Framework for the Classification of Microbial Gene Activity States

**DOI:** 10.3389/fmicb.2016.01191

**Published:** 2016-08-09

**Authors:** Craig Disselkoen, Brian Greco, Kaitlyn Cook, Kristin Koch, Reginald Lerebours, Chase Viss, Joshua Cape, Elizabeth Held, Yonatan Ashenafi, Karen Fischer, Allyson Acosta, Mark Cunningham, Aaron A. Best, Matthew DeJongh, Nathan Tintle

**Affiliations:** ^1^Department of Mathematics, Statistics and Computer Science, Dordt CollegeSioux Center, IA, USA; ^2^Department of Biostatistics, School of Public Health, University of MichiganAnn Arbor, MI, USA; ^3^Department of Statistics, University of TexasAustin, TX, USA; ^4^Department of Biostatistics, Harvard UniversityBoston, MA, USA; ^5^Department of Statistics, Baylor UniversityWaco, TX, USA; ^6^Department of Mathematics, University of DenverDenver, CO, USA; ^7^Department of Applied Mathematics and Statistics, Johns Hopkins UniversityBaltimore, MD, USA; ^8^Department of Biostatistics, University of IowaIowa City, IA, USA; ^9^Department of Statistics, Texas A&M UniversityCollege Station, TX, USA; ^10^Department of Computer Science, Hope CollegeHolland, MI, USA; ^11^Department of Biology, Hope CollegeHolland, MI, USA

**Keywords:** metabolic modeling, gene expression, bacteria, gene activity, Bayesian model

## Abstract

Numerous methods for classifying gene activity states based on gene expression data have been proposed for use in downstream applications, such as incorporating transcriptomics data into metabolic models in order to improve resulting flux predictions. These methods often attempt to classify gene activity for each gene in each experimental condition as belonging to one of two states: *active* (the gene product is part of an active cellular mechanism) or *inactive* (the cellular mechanism is not active). These existing methods of classifying gene activity states suffer from multiple limitations, including enforcing unrealistic constraints on the overall proportions of active and inactive genes, failing to leverage *a priori* knowledge of gene co-regulation, failing to account for differences between genes, and failing to provide statistically meaningful confidence estimates. We propose a flexible Bayesian approach to classifying gene activity states based on a Gaussian mixture model. The model integrates genome-wide transcriptomics data from multiple conditions and information about gene co-regulation to provide activity state confidence estimates for each gene in each condition. We compare the performance of our novel method to existing methods on both simulated data and real data from 907 *E. coli* gene expression arrays, as well as a comparison with experimentally measured flux values in 29 conditions, demonstrating that our method provides more consistent and accurate results than existing methods across a variety of metrics.

## Introduction

Numerous approaches to understanding and utilizing gene expression measurements attempt to classify them into one of two states: *active* (roughly speaking, the gene product is part of an active cellular mechanism) or *inactive* (the cellular mechanism is not active) (Ferrell, 2002; Abel et al., 2013; Gallo et al., 2015). We label this classification a determination of the *gene activity state*. These approaches are becoming more and more relevant with continued dramatic increases in the quantity and diversity of transcriptomics data as prices to obtain data continue to decline. In particular, recent approaches to metabolic modeling (MM) have focused on the integration of multiple sources of genetic information including transcriptomics data (Pfau et al., 2011; Lewis et al., 2012; Bordbar et al., 2014; Chubukov et al., 2014; Machado and Herrgård, 2014; Monk et al., 2014; Rezola et al., 2014). In these approaches, gene activity states are usually incorporated into constraints on the fluxes through reactions associated with the gene products. For example, GIMME (Becker and Palsson, 2008) applies a user-specified expression level threshold to classify gene activity states in any given experiment, then computes a penalty for flux through any reaction associated with an inactive gene; Flux Balance Analysis (FBA) is then constrained by minimizing the sum of penalties across all reactions in the model. PROM (Chandrasekaran and Price, 2010) uses a version of this approach in which the researcher finds the user-specified expression level threshold by assuming that a pre-defined percentage of all genes in an experiment are active (e.g., 33% Chandrasekaran and Price, 2010 or 50% Dunman, Personal Communication.). Others have proposed approaches in a similar spirit (Jerby and Ruppin, 2012), while some have allowed for more uncertainty through the addition of an “*unsure”* state, which yields no corresponding flux constraint (Shlomi et al., 2008), or by examining relative expression values (Jensen and Papin, 2011). Some (Jensen et al., 2011) suggest that large relative changes in gene expression (above a threshold) signal a shift from one state to the other, while others (Van Berlo et al., 2011) use both absolute and relative changes. Most recently, some have proposed continuous approaches whereby larger expression values for an experiment are classified as more likely to be active, and lower expression values for an experiment are classified as less likely to be active (e.g., GIM3E, Schmidt et al., 2013). Constraints on FBA via the estimated gene expression states are “soft” in that they can be violated to allow for uncertainty in expression state classification and also allow for potential post-transcriptional control; precise handling of such violations varies among approaches but typically involves a penalty term in the linear programming problem. While PROM classifies gene states in the standard manner, PROM does not directly constrain FBA based on the states.

While not all MM approaches to integration of transcriptomics data attempt to classify gene activity measurements into two states (Colijn et al., 2009; Moxley et al., 2009; Fang et al., 2012; Kim and Reed, 2012; Lee et al., 2012; Navid and Almaas, 2012), integrated MM approaches which do classify gene states suffer from at least four major limitations. First, many of the existing methods do not allow for different activity state thresholds between genes (e.g., gene A is assigned a threshold of 9.25 on a log-scale between active and inactive states; whereas gene B is assigned a threshold of 10). Second, many existing methods do not allow differences in the proportion of genes that are active from one experiment to another (e.g., a higher proportion of genes are expected to be classified as active in a minimal media condition than in a rich media condition). Third, existing methods do not leverage *a priori* knowledge about potential gene co-regulation to improve activity state classification (e.g., given that two genes A and B are co-regulated, if A is classified as active, B should also be classified as active). Finally, almost all existing methods do not meaningfully estimate statistical uncertainty in the classification process; the typical approaches to classifying genes using Boolean rules (every gene is either active or inactive) do not attempt to incorporate uncertainty in the classification. While some have attempted to incorporate uncertainty into the classification process (e.g., some have an “unsure” classification Shlomi et al., 2008), all approaches (including PROM Chandrasekaran and Price, 2010) incorporate *post-hoc* uncertainty adjustments by allowing violations of the gene activity state using penalties which apply similarly across all genes—in essence uniformly down-weighting the impact of expression data to account for upstream processing uncertainty. Here we propose improvements to gene activity state classification that address these limitations. Our work is motivated by the observation that many researchers work under operational definitions of genes as “active” in some conditions and “inactive” in others. Our goal is to provide guidance to researchers who regularly put this intuition into practice, by assessing their methods for classifying genes into activity states based on gene expression data, and proposing statistical models for data analysis that lead to improved classifications. Thus, we propose a flexible Bayesian approach that uses parametric mixture models as a platform for meaningfully estimating gene activity states through the integration of expression data and knowledge of operon structure. We quantify confidence in gene state estimates, which subsequently then can be incorporated into downstream analyses. We assess the performance of the model against other common approaches of estimating gene activity states using both simulated data and real *E. coli* transcriptome data; we compare our activity estimates to predictions of gene activity derived from reaction fluxes in a metabolic model of *E. coli* as well as to experimentally measured reaction fluxes in *E. coli* (Ishii et al., 2007; Machado and Herrgård, 2014).

## Methods

### General mixture modeling framework

Throughout this paper, we consider a set of *m* bacterial genes from a single organism whose expression levels ϵ have been observed across *n* different experimental settings. We define ϵ_*ij*_ to be the expression level recorded for the *i*th gene in the *j*th experimental sample, where ϵ_*ij*_ is the background corrected, normalized, logarithm of recorded amount of mRNA from an expression array. In the spirit of Becker and Palsson (2008) and Chandrasekaran and Price (2010), for each gene, *i*, we consider these observed ϵ_*ij*_ values to come from one of two possible (unobserved) gene states: inactive (gene *i* is producing only basal levels of product) and active (gene *i* is producing product involved in a functioning cellular process).

A natural probabilistic model for the observed gene expression levels for genes in each state is a conditional Gaussian model, which follows earlier work in other genomic contexts (Gamba et al., 2015; Morfopoulou and Plagnol, 2015). Namely, ϵ_*i*_|*active* ~ *N*(μ_1_, σ_1_) and ϵ_*i*_|*inactive* ~ *N*(μ_0_, σ_0_). In other words, the distribution of expression values for a gene in the active state follows a Gaussian distribution with an underlying mean, μ_1_, and standard deviation, σ_1_ where the standard deviation captures the underlying measurement (Ohtaki et al., 2010) and biological variability (Losick and Desplan, 2008; Chalancon et al., 2012) in expression measurements across settings. Similar assumptions and definitions hold for gene expression measurements from the inactive state. Since in most settings, the true state of the gene is unknown *a priori*, the resulting observed gene expression values, ϵ_*i*_ = (ϵ_*i*1_, ϵ_*i*2_, ϵ_*i*3_, …, ϵ_*in*_), can be modeled as coming from a Gaussian mixture distribution, ϵ_*i*_ ~ (1 − π)*N* (μ_0_, σ_0_) + π*N*(μ_1_, σ_1_), where the mixing parameter π represents the proportion of the time that the given gene *i* is active across the set of experiments.

### Univariate inference overview

Our goal is to use ϵ_*i*_ = (ϵ_*i*1_, ϵ_*i*2_, ϵ_*i*3_, …, ϵ_*in*_), and the Gaussian mixture model described above to make inferential statements about α_*i*_ = (α_*i*1_, α_*i*2_, α_*i*3_, …, α_*in*_), where α_*ij*_ ∈ {0, 1} indicates whether gene *i* = *1,… m* is in the active state (α_*ij*_ = 1) or the inactive state (α_*ij*_ = 0) in experimental setting *j* = *1,…,n*. In particular, since we cannot observe α_*i*_ directly, we wish to generate *a*_*i*_ = (*a*_*i*1_, *a*_*i*2_, *a*_*i*3_, …, *a*_*in*_), such that *a*_*ij*_ is the posterior probability that gene *i* is active in experiment *j*. We will use a Bayesian approach to generate *a*_*i*_ for each gene *i*.

For each gene *i*, we start with prior distributions on the five unknown parameters from the Gaussian mixture model: π, μ_0_, σ_0_, μ_1_, σ_1_. In practice, we reduce to four unknown parameters by requiring σ_0_ = σ_1_. This assumption provides increased model convergence and robustness to outliers (Fraley and Raftery, 2007), and assumes that similar amounts of biological and measurement variability will be present in expression values for the inactive and active states. The prior distributions for each of the four unknowns are given as: $\mu_{0}\;\sim \;N\left(\mu =8,\sigma =\sqrt{3}\right)$, $\mu_{1}\sim N\left(\mu =9,\sigma =\sqrt{3}\right)$, ${\sigma_{0}}^{2}={\sigma_{1}}^{2}\sim InverseWishart\left(\text{Ψ}=3,\nu =1\right)$ and π ~ *Beta*(α = 5, β = 5). Briefly, these choices of prior distributional shapes make later mathematical computation of posterior distributions straightforward and are standard in statistical practice (Murphy, 2007). The corresponding parameter values reflect reasonable experimental and biological assumptions [e.g., *E*(π) = 0.5; *E*(μ_0_) < *E*(μ_1_), etc.]. The choices of 8 and 9 for the prior means of RMA normalized data (See Section Real Data Sets), represent values near the overall “average” ϵ across all genes and all experiments (the range of which tends to be between 4 and 16). We note that, for this application, our analysis of the robustness of parameter choices indicates that these choices appear to have little bearing on resulting downstream *a*_*ij*_ generation (detailed results not shown).

We use a Gibbs sampler to generate *a*_*i*_ as follows:

Step 1. Let ${\hat{\mu }}_{0,k=1}=E\left(\mu_{0}\right)=8,{\hat{\mu }}_{1}=E\left(\mu_{1,k\;=\;1}\right)=9,\;{\hat{\sigma }}_{0,k\;=\;1}={\hat{\sigma }}_{1,k\;=\;1}=E\left(\sigma_{0}\right)=1,$ and ${\hat{\pi }}_{k\;=\;1}=E\left(\pi \right)=0.5$, where k = 1 indicates that this is the initial pass through the Gibbs sampler.

Step 2. Use the four estimated parameter values from Step 1 to find the estimated Gaussian mixture model ${\hat{\epsilon }}_{i,k\;=\;1}\sim \left(1-{\hat{\pi }}_{k\;=\;1}\right)N\left({\hat{\mu }}_{0,k\;=\;1},{\hat{\sigma }}_{0,k\;=\;1}\right)\;+\;{\hat{\pi }}_{k=1}N\left({\hat{\mu }}_{1,k=1},{\hat{\sigma }}_{1,k=1}\right)$.

Step 3. Use the estimated Gaussian mixture model, ${\hat{\epsilon }}_{i,k=1}$, to find *b*_*ij, k* = 1_(ϵ_*ij*_), the conditional probability that an expression value, ϵ_*ij*_, is from the active state, where $b_{ij,k=1}=\frac{{\hat{\pi }}_{k=1}f_{1,k=1}\left(\epsilon_{ij}\right)}{{\hat{\pi }}_{k=1}f_{1,k=1}\left(\epsilon_{ij}\right)\;+\;\left(1-{\hat{\pi }}_{k=1}\right)f_{0,k\;=\;1}\left(\epsilon_{ij}\right)}$ where $f_{1,k=1}(\epsilon_{ij})=\frac{1}{{\hat{\sigma }}_{1,k=1}\sqrt{2\pi }}e^{-\frac{{\left(\epsilon_{ij}-{\hat{\mu }}_{1,k=1}\right)}^{2}}{2{\hat{\sigma }}_{1}^{2}{}_{,\;k=1}}}$ and $f_{0,k\;=\;1}(\epsilon_{ij})=\frac{1}{{\hat{\sigma }}_{0,k\;=\;1}\sqrt{2\pi }}e{\;}^{-\frac{{\left(\epsilon_{ij}-{\hat{\mu }}_{0,k\;=\;1}\right)}^{2}}{2{\hat{\sigma }}_{0}^{2},\;k\;=\;1}}$

Step 4. Generate a random vector *I*_*i, k* = 1_, where *I*_*ij, k* = 1_is a single random value {0 or 1} drawn from *Bernoulli*(*p* = *b*_*ij, k* = 1_), indicating whether gene *i* is active or inactive in experimental setting *j*, for the *k* = 1 iteration of the Gibbs sampler.

Step 5. Update the prior distributions of the four parameters by incorporating the prior distributions with *I*_*i, k* = 1_. In particular, let *C*_*active*_ and *C*_*inactive*_ be the set of expression values currently assigned to the active and inactive clusters, respectively, according to *I*_*i*_. Then $\mu_{0}\sim \;N({\left(n\sigma_{0}{}^{-2}+{\sqrt{3}}^{-1}\right)}^{-1}(8{\sqrt{3}}^{-1}+n\sigma_{0}{}^{-2}{\bar{\epsilon }}_{0})$, ${\left(n\sigma_{0}{}^{-2}+{\sqrt{3}}^{-1}\right)}^{-1})$, $\mu_{1}\sim N({\left(n\sigma_{1}{}^{-2}+{\sqrt{3}}^{-1}\right)}^{-1}(9{\sqrt{3}}^{-1}+n\sigma_{1}{}^{-2}\bar{\epsilon_{1}})$, ${\left(n\sigma_{1}{}^{-2}+{\sqrt{3}}^{-1}\right)}^{-1})$, $\sigma_{0}{}^{2}=\sigma_{1}{}^{2}\sim InverseWishart(\Psi +\underset{j\in C_{active}}{\sum^{\text{​}}}{\left(\epsilon_{ij}-\mu_{1}\right)}^{T}\;(\epsilon_{ij}-\mu_{1})+\;\underset{j\;\in \;C_{inactive}}{\sum^{\text{​}}}{\left(\epsilon_{ij}-\mu_{0}\right)}^{T}(\epsilon_{ij}-\mu_{0}))\;\nu \;+\;n)$ and π ~ *Beta*(α + |*C*_*active*_|, β + |*C*_*inactive*_|), where |*C*_*active*_| represents the cardinality (size) of the set of expression values in *C*_*active*_. And, where ${\bar{\epsilon }}_{0}$ and ${\bar{\epsilon }}_{1}$ are the means of the classified inactive and active gene expression data, respectively.

The Gibbs sampler then repeats Steps 2–5 for *k* = *K* times. In our case, we used *K* = 500, with values less than 500 tending to give less robust results (detailed results not shown).

To generate *a*_*i*_, values of *I*_*i, k*_ are averaged across the *K* runs of the Gibbs sampler, ignoring an initial set of burn-in runs, *b*. In our case we used *b* = 50, which yielded robust *a*_*i*_ values (detailed results not shown). In particular, $a_{ij}=\frac{\overset{K}{\underset{k\;=\;b}{\sum }}I_{ij,k}}{K-b}$, for all *j*.

### Multivariate inference overview

While the univariate Gaussian mixture model and associated Gibbs sampler provide a standard way to generate *a*_*i*_ values gene by gene, this approach fails to account for other *a priori* known biological information which may be able to further improve *a*_*i*_ estimates. For example, in bacteria, operons are sets of contiguous genes that are co-regulated and therefore are generally active or inactive simultaneously. Thus, knowledge about which genes are in operons should allow us to improve gene activity estimates.

If there are *p* genes (*i*_1_*,i*_2_*,…,i*_*p*_) located within a given operon, *r*, then we can extend the univariate Gaussian mixture model described earlier to a multivariate Gaussian mixture model as follows:

$$\begin{array}{l} \epsilon_{r}=\epsilon_{i_{1},i_{2},\ldots ,i_{p}}\sim \left(1-\pi \right)N\left({\vec{\mu }}_{0},\Sigma_{0}\right)+\pi N\left({\vec{\mu }}_{1},\Sigma_{1}\right), \end{array}$$

Where, ${\vec{\mu }}_{0}=\left(\mu_{0,i_{1}},\mu_{0,i_{2}},\ldots ,\mu_{0,i_{p}}\right),{\vec{\mu }}_{1}=\left(\mu_{1,i_{1}},\mu_{1,i_{2}},\ldots ,\mu_{1,i_{p}}\right)$, ${\Sigma_{0}}^{2}=\left(\begin{array}{ccc} \sigma_{0,i_{1}}^{2} & \cdots & 0\\ \vdots & \ddots & \vdots \\ 0 & \cdots & \sigma_{0,i_{p}}^{2} \end{array}\right)$ and ${\Sigma_{1}}^{2}=\left(\begin{array}{ccc} \sigma_{1,i_{1}}^{2} & \cdots & 0\\ \vdots & \ddots & \vdots \\ 0 & \cdots & \sigma_{1,i_{p}}^{2} \end{array}\right)$, where we assume that within each state (active or inactive) the biological and measurement co-variability between gene expression measurements is zero.

Our approach in the multivariate case is very similar to our approach in the univariate case, and so is only outlined here. For each operon *r*, we start with prior distributions on the four unique and unknown parameters/vectors from the Gaussian mixture model: $\pi ,{\vec{\mu }}_{0},{\vec{\mu }}_{1},\Sigma_{0}=\Sigma_{1}$. The prior distributions for each of the four unknowns are given as: ${\vec{\mu }}_{0}\sim N\left(\vec{\mu }=\vec{8},\Sigma_{{\vec{\mu }}_{0}}=\left(\begin{array}{ccc} \sqrt{3} & \cdots & 0\\ \vdots & \ddots & \vdots \\ 0 & \cdots & \sqrt{3} \end{array}\right)\right)$, ${\vec{\mu }}_{1}\sim N\left(\vec{\mu }=\vec{9},\Sigma_{{\vec{\mu }}_{1}}=\left(\begin{array}{ccc} \sqrt{3} & \cdots & 0\\ \vdots & \ddots & \vdots \\ 0 & \cdots & \sqrt{3} \end{array}\right)\right)$,${\Sigma_{0}}^{2}={\Sigma_{1}}^{2}\sim InverseWishart\left(df=p+2,scale=\left(\begin{array}{ccc} 1 & \cdots & 0\\ \vdots & \ddots & \vdots \\ 0 & \cdots & 1 \end{array}\right)\right)$ and π ~ *Beta*(5, 5). These distributions and initial prior parameter values are mainly explained above. Off-main diagonal values of 0 in $\Sigma_{{\vec{\mu }}_{0}}\text{and}\Sigma_{{\vec{\mu }}_{1}}$ suggest that there is no correlation between the means of the active (or inactive) state distributions across the genes in an operon, with Σ_0_ = Σ_1_ suggesting no co-variability in the state expression variances of genes in an operon. As before, our analysis of the robustness of parameter choices indicates that these choices appear to have little bearing on resulting downstream *a*_*ij*_ generation (detailed results not shown). The Gibbs sampler is performed as described above by simply replacing the univariate parameters with the multivariate parameters. Notably, this yields *a*_*i*_ values which are identical across all *p* genes located within an operon, since inference about activity states is occurring for the operon as a whole, not gene-by gene.

### Implementation details for all methods being compared

In order to compare the performance of the two methods proposed above to current best practices, we implemented five approaches to estimating *a*_*ij*_: median thresholding, trichotomous thresholding, rank based estimation, and our proposed univariate mixture modeling and multivariate mixture modeling approaches. We now briefly describe the methods compared here, along with some relevant implementation notes:

#### Median threshold (MT)

This approach dichotomizes expression values such that *a*_*ij*_ = $\left\{\begin{array}{l} 0\;if\;\epsilon_{ij}<M_{j}\\ 1\;if\;\epsilon_{ij}\geq M_{j} \end{array}\right\}$, where *M*_*j*_ is median(ϵ_*ij*_) for all *m* genes in experiment *j*. This approach is a special case of that proposed by GIMME (Becker and Palsson, 2008), which allows users to, *a priori*, select any threshold (median or otherwise). In practice, some users select the median (**?**). We note that the GIMME software program uses the mean as the default value for the threshold^1^. Due to the typical symmetry of gene expression values within an experiment, the mean is nearly identical to the median.

#### Trichotomous threshold (TT)

This approach trichotomizes expression values such that $a_{ij}\;=\;\left\{\begin{array}{l} \begin{array}{c} \;0\;if\;\epsilon_{ij}\;<\;P_{low,j}\\ \text{0}.\text{5 }if\;P_{low,j}\;<\;\epsilon_{ij}\leq P_{high,j} \end{array}\\ \;1\;if\;\epsilon_{ij}\geq P_{high,j} \end{array}\right\}$, where *P*_*low, j*_ is the 40th percentile for all i genes in experiment j and *P*_*high, j*_ is the 60th percentile for all i genes in experiment j and is in the spirit of GIMME, but allowing for an uncertain region as proposed by Shlomi et al. (2008).

#### Rank based approach (RB)

This approach is a continuous analog to the *MT* approach. In particular, $a_{ij}=\frac{rank(\epsilon_{ij})}{m},$ where *rank*(ϵ_*ij*_) is the rank within experiment *j*. This approach is in the spirit of GIM3E (Schmidt et al., 2013) which assigns the equivalent of *a*_*ij*_ = 1 to *max*(ϵ_*ij*_), and a monotone and continuously changing decreasing confidence as ϵ_*ij*_ decreases.

#### Univariate mixture model (UniMM)

This approach, described above, uses a Bayesian approach to infer *a*_*ij*_ values according to a Gaussian mixture model.

#### Multivariate mixture model (MultiMM)

This approach, described above, uses a Bayesian approach to infer *a*_*ij*_ values according to a Gaussian mixture model while incorporating knowledge of operon structure.

### Screening and imputation methods for mixture model approaches

When implementing *UniMM* and *MultiMM* we first assessed the quality of the fit of a 2-component mixture model to the observed expression data for each gene or operon. In particular, some genes/operons may not change states (between active and inactive) across the available set of expression values (all *n* experiment settings), thus making a 2-component mixture distribution invalid. With this in mind we used a screening method to determine which genes/operons had strong evidence that they were 1-component instead of 2-component.

The screening method uses the Bayesian Information Criterion (*BIC*) to assess the fit of a 1-component (univariate or multivariate) Gaussian mixture distribution vs. a 2-component mixture distribution using the *R* package *Mclust*^2^. Following Raftery et al. (Raftery, 1995) we require the BIC to be at least 12 points better for the 1-component model to be chosen vs. the 2-component model. We note, however, that if we were to simply choose the best BIC between the 1 and 2 component models there would be little impact on the number of genes screened as being from a single component (detailed results not shown).

The *a*_*ij*_ values for genes which were screened as coming from only a single component (all active or all inactive) were estimated using a multiple imputation approach (*MI*) in order to identify similar genes/operons and impute *a*_*ij*_ values. Multiple imputation is a well-known statistical procedure for estimation of missing values (Rubin, 1987). When a gene, *i*, was screened as being from a single component, we used the R package *Mclust*^2^ to fit a single component Gaussian distribution to the data and estimate the corresponding mean and standard deviation of the model. Results of the *UniMM* approach for all genes from 2-component mixtures were then evaluated to identify “similar” genes, where similar genes had $\left({\hat{\mu }}_{0}\in {\bar{x}}_{\epsilon_{i}}\pm 0.1\;and\;{\hat{\sigma }}_{0}\in s_{\epsilon_{i}}\pm 0.1\right)$ or $\left({\hat{\mu }}_{1}\in {\bar{x}}_{\epsilon_{i}}\pm 0.1\;and\;{\hat{\sigma }}_{1}\in s_{\epsilon_{i}}\pm 0.1\right)$, where 0.1 is an arbitrary threshold. The multiple imputation approach then computes *a*_*i*_'s for each of the similar genes as if the ϵ_*i, j*_ came from each of the similar genes. The final *a*_*i*_'s for each imputed gene are computed by averaging across the imputed *a*_*i*_'s from each similar gene. In the case of operons identified as coming from a single component (*MultiMM* approach), each gene in the operon is first considered separately using the same *MI* approach as for the *UniMM* method, and then the resulting *a*_*i*_'s for each gene in the operon are averaged in order to yield consistent *a*_*i*_ values for all genes in the operon. For single component operons which are identified as always active or inactive, $\hat{\pi }=1\text{ or }0,$ respectively. Finally, we note that if no similar genes are identified, then the *MI* approach returns *a*_*ij*_ = 0.5 for all *j*.

### Real data sets

For most of our analyses, we used genome-wide gene expression data from 907 different microarray data sets collected on 4329 *Escherichia coli* genes via the M3D data repository (Faith et al., 2007, 2008,^3^). Raw data from Affymetrix^4^ CEL files were normalized using RMA (Irizarry et al., 2003). Details of data processing are described elsewhere (Tintle et al., 2012; Powers et al., 2015). We also performed analysis on gene expression and fluxomics data from Ishii et al. (Ishii et al., 2007) comprising 79 *E. coli* genes in 29 experimental conditions. *E. coli* operon predictions for 2648 operons, including 1895 single gene operons, were obtained from Microbes Online (Price et al., 2005).

### Simulated data sets

We also simulated expression data with “known” gene activity states (active/inactive). The simulation of expression data was informed by the *E. coli* expression data described above. We first ran the Screening Method described above (see Section Screening and Imputation Methods for Mixture Model Approaches) and dropped all operons, including single gene operons, for which the two-component model did not yield the highest BIC (*n* = 697 dropped). We then randomly selected 26.3% (= 697/2648) of the remaining 1951 operons to be single component in the simulated data, with each of the single component operons having an equal likelihood of being always active or always inactive.

We used two different methods to calculate the mixing parameter, π, used in the simulation for the 1438 two-component operons. The *Uniform Method* (*Unif*) chooses a random value for π between 0.2 and 0.8. The *Fitted Method* (*Fit*) uses the *MultiMM* estimate of π (details given above). Values for ${\vec{\mu }}_{0},{\vec{\mu }}_{1}$, Σ_0_ = Σ_1_ are all as estimated by the *MultiMM* method computed on the real expression data. To generate simulated expression values, $\epsilon_{ij}^{s}$, we drew 907(π_*i*_) random values from a multivariate normal distribution (${\vec{\mu }}_{1i},\Sigma_{1i}$) and 907(1 − π_*i*_) random values from a multivariate normal distribution (${\vec{\mu }}_{0i}$,Σ_0*i*_). Thus, we generated a 907 by 3435 matrix of $\epsilon_{ij}^{s}$ values.

### Validation of real gene activity calls using flux variability analysis

In order to generate alternative predictions of gene activity we used Flux Variability Analysis (Mahadevan and Schilling, 2003) on the *E. coli* iJO1366 metabolic model (Orth et al., 2011). In particular, we ran flux variability analysis on the *E. coli* metabolic model yielding flux bounds *v*_*low*_ and *v*_*high*_ for each reaction in the model. Media conditions and gene mutations were accounted for in a model maximizing biomass. The following rules were then used to determine predictions *r*_*ij*_ of reaction activity for each reaction in the model and each of the 907 experiments.

$$\begin{array}{l} r_{ij}=\left\{\begin{array}{c} 0\;if\;v_{ij,low}=0\\ 1\;if\;v_{ij,low}\;>\;0 \end{array}\right\} \end{array}$$

These reaction-level predictions were converted to gene-level predictions, *p*_*ij*_, accounting for isozymes and multi-gene complexes as follows. If *r*_*ij*_ = 1 and the reaction is associated with a single gene or multi-gene complex, *p*_*ij*_ for all genes involved is 1. If *r*_*ij*_ = 0 and the reaction is associated with a single gene or multiple isozymes, *p*_*ij*_ for all genes involved is 0. If *r*_*ij*_ = 1 but the reaction is associated with multiple isozymes, we cannot be sure which isozyme is responsible for enabling the reaction, so we make no prediction (assign no *p*_*ij*_ value) for any of the genes involved. If *r*_*ij*_ = 0 but the reaction is associated with a multi-gene complex, we cannot be sure which subunit is responsible for thwarting reaction activity, so we make no prediction (assign no *p*_*ij*_ value) for any of the genes involved. If the previous four rules result in contradictory *p*_*ij*_ values for any given gene (e.g., both *p*_*ij*_ = 0 and *p*_*ij*_ = 1), then we let *p*_*ij*_ = 1 for that gene. This assumes that a gene with multiple roles can be active but perform only some of its roles; for instance, a gene product may be associated with an active reaction, but also be an isozyme on an inactive reaction, resulting in contradictory *p*_*ij*_ values. In these cases *p*_*ij*_ = 1 is the correct prediction. Finally, we note that all but three of the 907 experiments resulted in a growth prediction by the metabolic model, and that, following these rules, *p*_*ij*_ values could be obtained for approximately 845,000 of the 1.2 million gene-by-experiment combinations in the metabolic model.

### Statistical analysis

We used two primary approaches to evaluate the quality of *a*_*ij*_'s resulting from different methods applied to both simulated and real data. The *squared deviation approach* quantifies the difference between *a*_*ij*_'s and true or predicted gene activity states. We computed $d_{ij}^{2}={\left(a_{ij}-\alpha_{ij}\right)}^{2}$ for simulated data, where α_*ij*_ is the true gene activity state, and $d_{ij}^{2}={\left(a_{ij}-p_{ij}\right)}^{2}$ for the real expression data where *p*_*ij*_ is the predicted gene activity state based on the flux variability analysis of the metabolic model. We note that *d*_*ij*_ is only computed on the 1353 genes in the metabolic model for the real expression data, since other genes have no *p*_*ij*_ values. The average deviation can then be computed by experiment, by gene or for various other subsets of the data.

The *consistency approach* indicates that an *a*_*ij*_ is consistent with *p*_*ij*_ or α_*ij*_ if a dichotomized version of the *a*_*ij*_ is consistent with *p*_*ij*_ or α_*ij*_. In particular, *c*_*ij*_ is computed as follows:

$$\begin{array}{lll} c_{ij} & = & \left\{\begin{array}{c} \begin{array}{c} 1\;if\;a_{ij}\;>0.5\;and\;p_{ij}\;=1\\ 0\;if\;a_{ij}\;>0.5\;and\;p_{ij}\;=0 \end{array}\\ 0\;if\;a_{ij}\;<0.5\;and\;p_{ij}\;=1\\ \begin{array}{c} 1\;if\;a_{ij}\;<0.5\;and\;p_{ij}\;=0\\ 0.5\;if\;a_{ij}\;=0.5 \end{array} \end{array}\right\}\text{or}\\ c_{ij} & = & \left\{\begin{array}{c} \begin{array}{c} 1\;if\;a_{ij}\;>0.5\;and\;\alpha_{ij}\;=1\\ 0\;if\;a_{ij}\;>0.5\;and\;\alpha_{ij}\;=0 \end{array}\\ 0\;if\;a_{ij}\;<0.5\;and\;\alpha_{ij}\;=1\\ \begin{array}{c} 1\;if\;a_{ij}\;<0.5\;and\;\alpha_{ij}\;=0\\ 0.5if\;a_{ij}\;=0.5 \end{array} \end{array}\right\} \end{array}$$

A consistency score can then be computed by summing *c*_*ij*_ across various subsets of the data (e.g., all genes within an experiment; all genes in an operon, etc.).

Lastly, we evaluated the differential expression of metabolic pathway components (DeJongh et al., 2007; Aziz et al., 2008; Henry et al., 2010) among different subsets of experiments using a modified gene set analysis approach (Tintle et al., 2008). These pathway components have been previously shown to demonstrate strong consistency with gene expression data (Tintle et al., 2012). Briefly, we found the average *a*_*ij*_ value for all genes within each pathway component in each of the 907 experiments, and then ran a two-sample *t*-test comparing the mean activity scores between different subsets of the 907 experiments.

### Validation using experimentally measured fluxes

Lastly, we evaluated gene activity estimates inferred from expression data vs. experimentally measured reaction fluxes on a published set of 79 genes in 29 separate experimental conditions (Ishii et al., 2007; Machado and Herrgård, 2014). In short, we computed gene activity estimates (*a*_*ij*_'s) for each gene-experiment combination using methods described above. After generating the gene-level *a*_*ij*_'s, we mapped them to reaction-level predictions *q*_*ij*_, accounting for isozymes and multi-gene complexes as follows. For each reaction, if the reaction is associated with a single gene, *q*_*ij*_ for that reaction is equal to the *a*_*ij*_ for that gene. If the reaction is associated with a multi-gene complex, *q*_*ij*_ for that reaction is equal to the minimum of the *a*_*ij*_'s for all the genes involved; and if the reaction is associated with isozymes, *q*_*ij*_ for that reaction is equal to the maximum of the *a*_*ij*_'s for all the genes involved. In any of these three cases, if any gene has no *a*_*ij*_ (because it was not one of the 79 genes for which expression data was measured), it is ignored for the purposes of taking the minimum or maximum; or if all of the genes associated with a given reaction have no *a*_*ij*_, that reaction is dropped from the analysis. We repeated this procedure with each *a*_*ij*_ generation method, and also with the gene-level ϵ_*ij*_'s for comparison purposes, to create alternate reaction-level predictions based directly on expression value.

We compared the correlations we observed between each set of *q*_*ij*_'s with the (absolute values of the) experimentally measured fluxes for those reactions in those experimental conditions. A square-root transformation was applied to both the fluxes and the ϵ_*ij*_-based *q*_*ij*_'s to normalize these skewed distributions to ensure robust correlation estimates were obtained. Multiple linear regression models were used to predict fluxes using gene activity method estimates and expression values in order to evaluate the explanatory ability of different gene activity state estimates with flux values.

### Software

*R* scripts and an example implementation of the approaches considered here (*MT, TT, RB, UniMM*, and *MultiMM*) are freely available on the Software page at http://www.dordt.edu/statgen.

## Results

### Performance on simulated data

**Figure 1:**
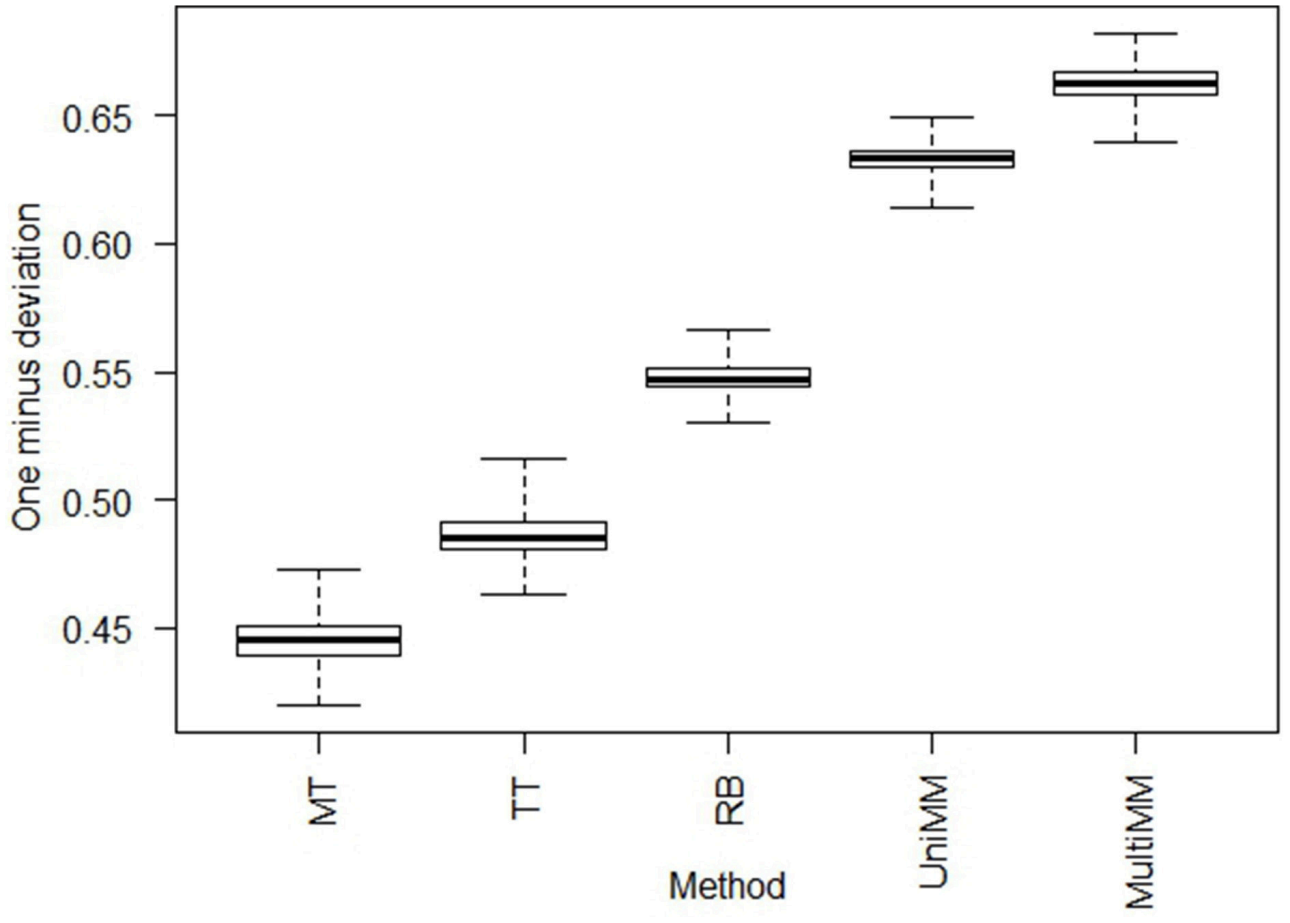
**Boxplots of deviations from true activity state by approach**. Boxplots represent the value of 1−*d*_*i*_ across each of the experiments, *i* = 1,…, 907, where $d_{i}=\sqrt{d_{i}^{2}}$ and $d_{i}^{2}=$ the average $d_{ij}^{2}$ across all genes *j* = *1,…,m* in experiment *i* (see Section Methods: Statistical analysis for details). Larger numbers on the y-axis represent less deviation from true activity states, illustrating that *MultiMM* has the best performance, followed by *UniMM*, with *MT* yielding the worst performance. This figure illustrates the results on simulated data using the *Unif* simulation approach. Performance with the *Fitted* approach was similar.

We begin by evaluating the performance of the different approaches to *a*_*ij*_ estimation on simulated data. Figure 1 illustrates that the *Univariate mixture model* (*UniMM*) and *Multivariate Mixture Model (MultiMM)* models tend to outperform the other previously proposed approaches [*Median Threshold (MT); Trichotomous Threshold (TT); Rank-based (RB)*] by yielding the least deviation from true gene activity states, with *MultiMM* performing best. Table 1 likewise shows the overall performance of the five approaches for the simulated data using the consistency metric. Both Mixture Model (*MM)* approaches yield the best sensitivity and best specificity, with the *MultiMM* method again performing best overall. The *MultiMM* method also performed best compared to other methods when examining only the subset of genes identified as coming from a single component. Table 2 illustrates that the *MM* approaches also give the most consistent results at different confidence levels. Finally, Table 3 shows similarly top performance of the *MultiMM* approach for operons, with the *MultiMM* approach yielding the most consistent calls with no inconsistencies, and the best overall concordance with the simulated data (85.3% consistent and correctly assigned compared to 58% or worse for all other approaches). Tables 1–3 are shown based on data from the *Unif* simulation approach. Results using the fitted simulation approach are similar (detailed results not shown).

**Table 1:** **Overall consistency^**a**^ of gene activity state approaches with simulated activity states**.

**Subset**	**Number of gene by experiment combinations (in thousands)**	**Method**				
		***MT* (%)**	***TT* (%)**	***RB* (%)**	***UniMM* (%)**	***MultiMM* (%)**
Among gene-experiment combinations the simulator assigned as active (*α_ij_* = 1; *Sensitivity*)	1569	69.1	68.4	69.1	76.5	81.4
Among gene-experiment combinations the simulator assigned as inactive (*α_ij_* = 0; *Specificity*)	1547	69.4	68.7	69.4	81.8	86.4
Sensitivity + Specificity^b^	–	138.5^c^	137.1	138.5^c^	158.3	167.8
Only data points from genes flagged as 2-component	2209	69.3	68.6	69.3	84.8	89.4
Only data points from genes flagged as 1-component^d^	907	69.2	68.4	69.2	65.3	70.3

a *Values in this table are reported as 100% times average consistency (c_ij_) across the indicated subset of the data (leftmost column)*.

b *Instead of maximizing the Sensitivity + Specificity, a researcher could choose to maximize the average c_ij_ across all 3,115,545 gene-experiment combinations. This would result in values as follows: 69.3% (MT), 68.6% (TT), 69.3% (RB), 79.2% (UniMM), and 83.9% (MultiMM), again demonstrating the benefit of the MM approaches*.

c *By definition these approaches will yield the same result since we are dichotomizing the RB a_ij_'s when computing c_ij_*.

d *These values are based on the genes flagged as one component by the MultiMM method; when using genes flagged by the UniMM method, results were comparable*.

**Table 2:** **Overall method consistency with simulated gene activity state assignments stratified by confidence**.

**Approach**	**Confidence level**		
	**High (0 ≤ *a_ij_* < 0.2; 0.8 < *a_ij_* ≤ 1)**	**Medium (0.2 ≤ *a_ij_* < 0.4; 0.6 < *a_ij_* ≤ 0.8)**	**Low (0.4 < *a_ij_* ≤ 0.6)**
MT	69.3% (2158/3116)^a^	−	−
TT	73.2% (1824/2429)	−	50% (312/624)
RB	80.9% (1009/1247)	65.5% (816/1246)	53.5% (333/622)
UniMM	93.4% (1623/1737)	65.4% (577/883)	53.7% (266/495)
MultiMM	95.3% (1849/1941)	70.1% (530/757)	55.8% (233/418)

a *For example, 2158 is the number of consistent gene-experiment combinations at high confidence for the MT approach (in thousands), and 3116 is the total number of gene-experiment combinations at high confidence for the MT approach (in thousands)*.

**Table 3:** **Method consistency within operons with simulated gene activity state assignments**.

**Approach**	**Inconsistent^a^ (at least one a *a_ij_* > 0.6 and one < 0.4)**	**Consistent (all *a_ij_* > 0.4 or *a_ij_* < 0.6)**	**Percent consistent and correct^b^ activity assignment**
MT	34.5% (207/599^c^)	65.5% (392/599)	50.1% (300/599)
TT	16.9% (101/599)	83.2% (498/599)	58.9% (353/599)
RB	16.9% (101/599)	83.0% (497/599)	50.1% (300/599)
UniMM	20.0% (120/599)	80.0% (479/599)	58.2% (349/599)
MultiMM	0% (0/599)	100% 599/599	85.3% (511/599)

a *Inconsistent occurs when one or more genes within the same operon are indicated likely to be active and one or more genes within that same operon are indicated likely to be inactive*.

b *Correct means that the consistent operon activity calls are also identified correctly as active or inactive (based on the underlying simulation model)*.

c *All counts in the table are reported in 1000s, representing the number of operon-experiment combinations*.

### Performance on real data

Table 4 provides the overall performance of each of the five approaches for inferring gene activity states as compared to metabolic model flux predictions. Overall, the *MM* approaches yielded better specificity, at the expense of sensitivity, by, in general, yielding fewer *a*_*ij*_ > 0.5 than the *MT, TT*, and *RB* methods. This resulted in a larger combined sensitivity plus specificity for the *MM* approaches, with a slight preference to the *MultiMM* method using this metric. Both *MM* approaches also yielded better *c*_*ij*_ values when evaluating genes flagged as one component. Table 5 illustrates the overall performance of each of the five approaches by confidence. *MM* approaches provide substantially improved consistency over the other approaches when confidence levels are high or medium, with similar performance for low certainty *a*_*ij*_ values. Sensitivity and specificity trends by confidence and approach follow directly from the patterns observed in Table 4 (detailed results not shown). Finally, Table 6 shows the consistency of *a*_*ij*_'s within operons. As noted in the table, the *MultiMM* method provides calls which are consistent within operons, which is expected based on the way that calls are made for each operon when using the *MultiMM* approach. We note that our analysis evaluating consistency of the gene activity state estimates with results from Flux Variability Analysis are limited by the quality of the modeling results from FVA and inherent limitations of the FVA approach. Thus, sensitivity and specificity estimates provided here should also not be viewed without recognizing these limitations.

**Table 4:** **Overall consistency^**a**^ of gene activity state approaches with metabolic model flux predictions**.

**Subset**	**Number of gene by experiment combinations (in thousands)**	**Method**				
		***MT* (%)**	***TT* (%)**	***RB* (%)**	***UniMM* (%)**	***MultiMM* (%)**
Among gene-experiment combinations the model predicted as active (*p_ij_* = 1; *Sensitivity*)	116	83.8	82.1	83.8	66.0	70.3
Among gene-experiment combinations the model predicted as inactive (*p_ij_* = 0; *Specificity*)	729	40.1	40.2	40.1	62.8	58.9
Sensitivity + Specificity^b^	–	123.9^c^	122.3	123.9^c^	128.8	129.2
Only data points from genes flagged as 2-component	721	45.3	45.2	45.3	62.6	59.4
Only data points from genes flagged as 1-component^d^	124	50.9	50.3	50.9	66.9	67.1

a *Values in this table are reported as 100% times average consistency (c_ij_) across the indicated subset of the data (leftmost column)*.

b *Instead of maximizing the Sensitivity + Specificity, a researcher could choose to maximize the average c_ij_ across all 844,807 gene-experiment combinations with p_ij_ predictions. This would result in values as follows: 46.1% (MT), 46.0% (TT), 46.1% (RB), 63.2% (UniMM), and 60.5% (MultiMM), again demonstrating the benefit of the MM approaches*.

c By definition these approaches will yield the same result since we are dichotomizing the RB a_ij_'s when computing c_ij_

d *These values are based on the genes flagged as one component by the MultiMM method; likewise, the values in the row above are based on the genes flagged as two component by the MultiMM method. When using genes flagged by the UniMM method, results were comparable*.

**Table 5:** **Overall method consistency vs. metabolic model predictions stratified by confidence**.

**Approach**	**Confidence level**		
	**High (0 ≤ *a_ij_* < 0.2; 0.8 < *a_ij_* ≤ 1)**	**Medium (0.2 ≤ *a_ij_* < 0.4; 0.6 < *a_ij_* ≤ 0.8)**	**Low (0.4 < *a_ij_* ≤ 0.6)**
MT	46.1% (390/845)^a^	−	−
TT	44.8% (295/658)	−	50.0% (94/187)
RB	39.2% (115/293)	49.4% (180/364)	50.6% (95/187)
UniMM	64.0% (368/574)	65.2% (117/179)	54.4% (50/92)
MultiMM	60.1% (395/656)	64.2% (77/120)	57.5% (39/68)

a *For example, 390 is the number of consistent gene-experiment combinations at high confidence for the MT approach (in thousands), and 845 is the total number of gene-experiment combinations at high confidence for the MT approach (in thousands)*.

**Table 6:** **Method consistency within operons**.

	**Consistency**		
**Approach**	**Very consistent (all > 0.8 or < 0.2)**	**Consistent (all > 0.4 or < 0.6, but not very consistent)**	**Inconsistent (at least one a *a_ij_* > 0.6 and one < 0.4)**
MT	66.6% 455/683	−	33.4% 228/683
TT	47.8% 327/683	35.7% 244/683	16.5% 113/683
RB	18.0% 123/683	65.5% 447/683	16.5% 113/683
UniMM	37.7% 258/683	43.3% 296/683	19.0% 130/683
MultiMM	79.8% 545/683	20.2% 138/683	0% 0/683

### Specific examples

#### L-arabinose operon

**Figure 2:**
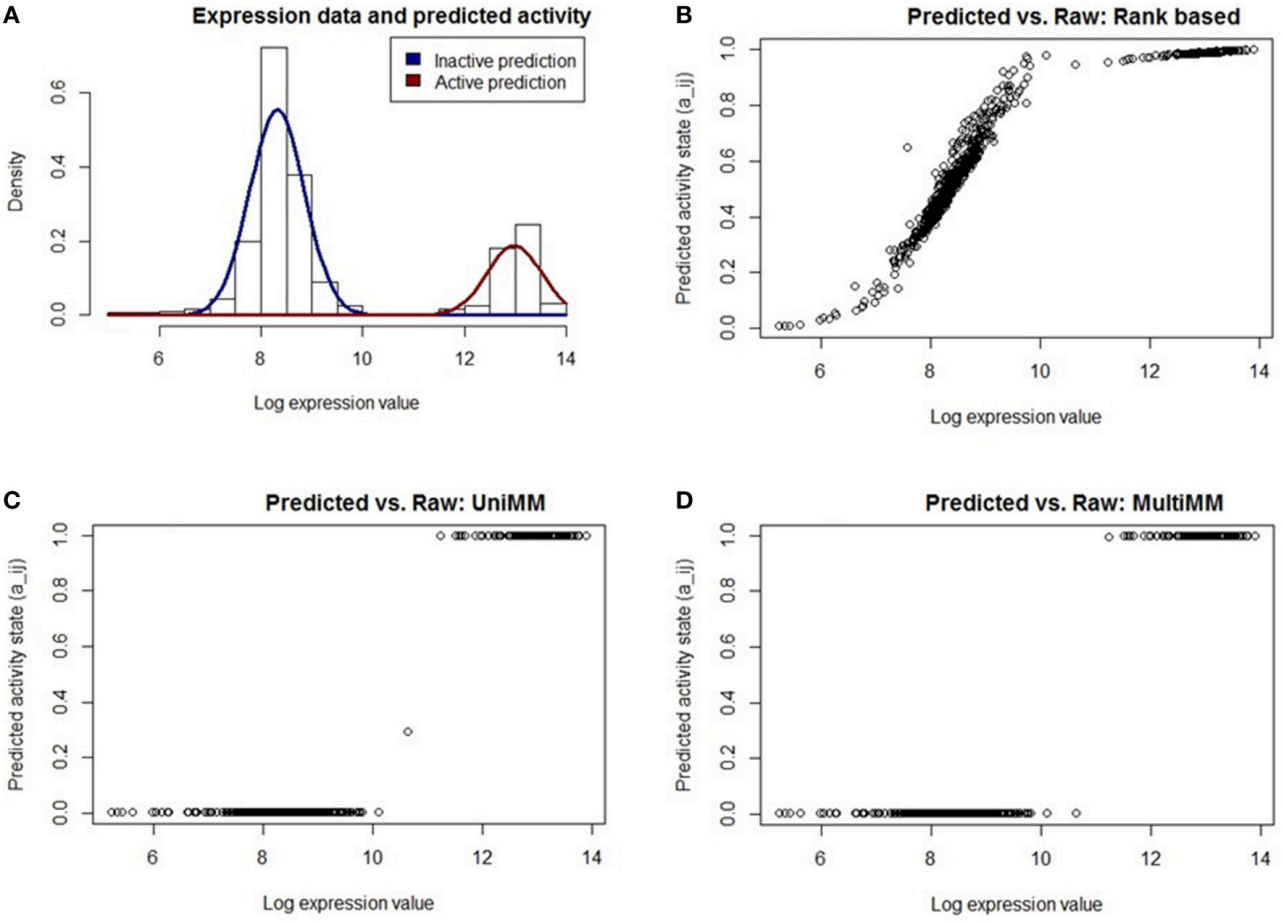
**Performance of different activity state inference methods on ***araB*****. Expression values are across 907 *E. Coli* experiments. **(A)** shows the raw expression data with an overlaid Gaussian mixture distribution from MultiMM for the *araB* gene. The remaining three figures **(B–D)** graph the posterior probability that *araB* is active vs. the log expression for each experiment using three different methods of generating posterior probabilities. The UniMM and MultiMM methods **(C,D)** yield results which more intuitively agree with the observed raw expression values than the rank-based approach **(B)**. The MultiMM method, by leveraging information from all genes in the operon, is able to provide improved certainty over the other methods.

**Figure 3:**
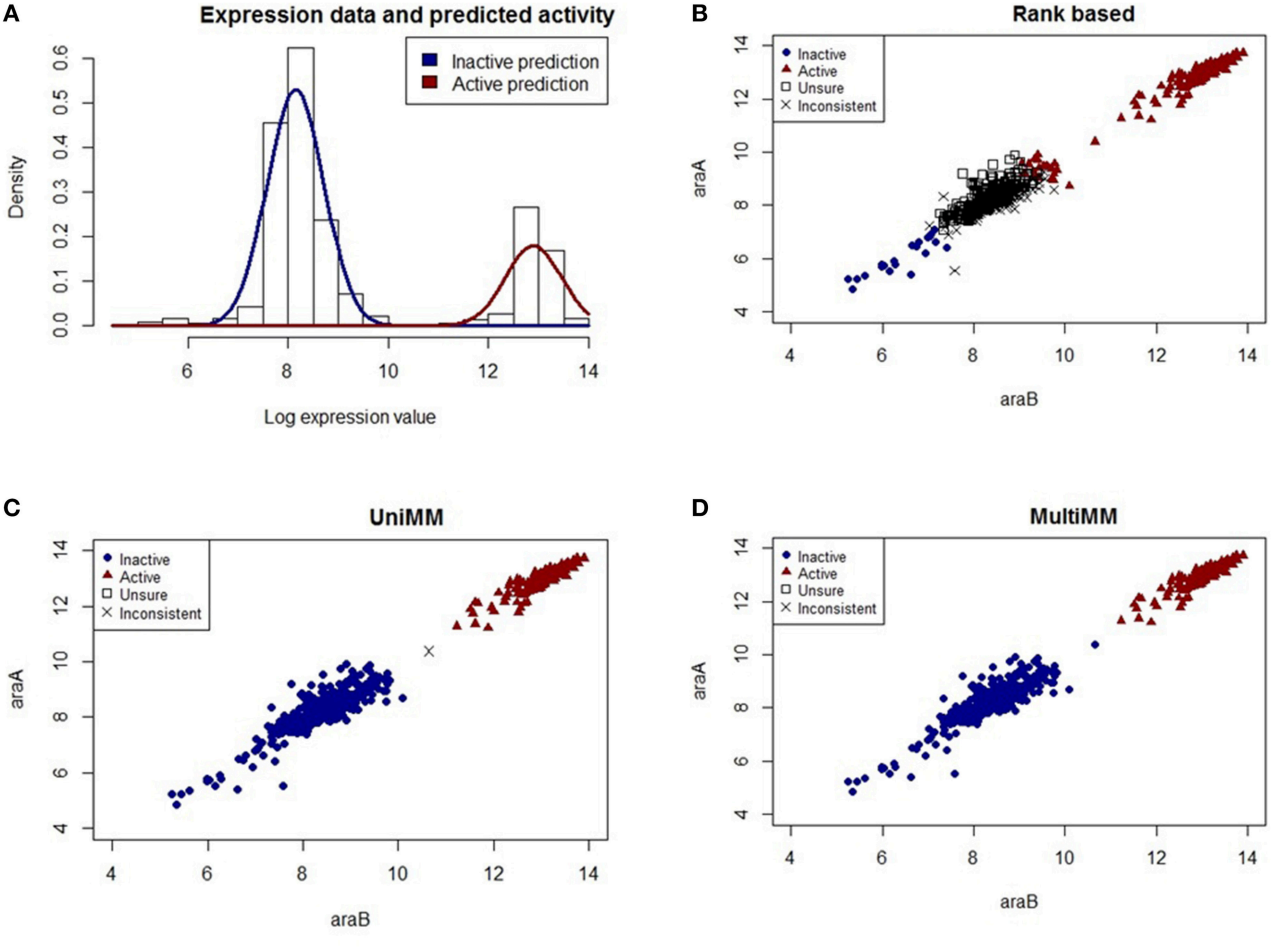
**Performance of different activity state inference methods on gene pair ***araB*** and ***araA***. (A)** shows the raw expression data with an overlaid Gaussian mixture distribution from the MultiMM method for the *araA* gene (the corresponding histogram for *araB* is in Figure 2A). **(B–D)** graph the observed expression values for *araA* vs. *araB* indicating how consistent or inconsistent the calls are for genes within an operon for each of three different methods of estimating gene activity states. Blue dots represent experiments for which the approach is very sure the gene pair is inactive (*a*_*ij*_ < 0.2) and red triangles represent experiments for which the approach is very sure the gene pair is active (*a*_*ij*_ > 0.8). Open squares represent unsure (0.2 < *a*_*ij*_ < 0.8) calls for both genes, and black “X's” represent situations where either *a*_*ij*_ < 0.2 for one gene and *a*_*ij*_ > 0.2 for the other, or *a*_*ij*_ < 0.8 for one gene and *a*_*ij*_ > 0.8 for the other. The MultiMM method, by leveraging information from all genes in the operon, is able to provide the most consistent calls of the three methods.

The L-arabinose (*ara*) operon is a well-studied set of three co-located genes (*araB, araA, araD*) which encode enzymes needed for the catabolism of arabinose in *E. coli* (Schleif, 2010). Across the 907 experiments in our dataset, the *MultiMM* algorithm calls the L-arabinose operon active (*a*_*ij*_ > 0.5) in 227 experiments and inactive in 680 experiments (*a*_*ij*_ < 0.5). In the vast majority of cases where the *MultiMM* identified the operon as active, L-arabinose was identified as present in the media (96.4% = 219/227), and all 8 inconsistent cases were from the same experimental series. Similarly, when our algorithm indicated that the *ara* operon was inactive, L-arabinose was not indicated as being present in the media in the vast majority of cases (94.9% = 645/680). Many of the inconsistent cases had reasonable biological explanations for why they appear inconsistent (see footnote B to Table 7). While the UniMM approach performed similarly, other approaches had substantially more inconsistencies between the presence of L-arabinose in the media and the activity state of the genes in the operon (e.g., 565 inconsistent experiments for MT, 353 for TT and 94 for RB). Figures 2, 3 further illustrate how the *MultiMM* performs better than other approaches for this operon.

**Table 7:** **MultiMM calls for the L-arabinose (***ara***) operon (***araB, araA, araD***)**.

**L-arabinose added to the media**	**Operon activity estimate (*a_ij_*)**	
	**Active (*a_ij_* > 0.5)**	**Inactive (*a_ij_* < 0.5)**
Yes	219	35^b^
No	8^a^	645
Total	227	680

a *All 8 experiments were from the same series of experiments (experimenter, lab, and condition), a series of experiments on wild-type E. coli in the presence of varying amounts of Norfloxacin. See Faith et al., 2007, Supplemental Table 4 experiments: WT_N0000_r[1,2], WT_N0025_r[1,2], WT_N0050_r[1,2], WT_N0075_r[1,2]*.

b *Of these 35 experiments, 8 were from mutant E. coli strains without the ara operon. (See Faith et al., 2007, Supplemental Table 4 experiments: pBAD_ryhB_with_ara_r[1,2], pNM12_with_ara_r[1,2], pBAD_ryhB_iron_with_ara_r[1,2], pNM12_iron_with_ara_r[1,2]), 15 were from time series experiments measured at time 0 (potentially before the bacteria had time to react to the presence of L-arabinose; See Faith et al., 2007, ccdB_K12_t0_r1, lacZ_K12_t0_r1, lacZ_MG1063_t0_r[1,2], ccdB_MG1063_t0_r[1,2], lacZ_W1863_t0_r1, ccdB_W1872_t0_r1, ccdB_chelator_W1872_t0_r1, lacZ_MG1655_t0_r1, ccdB_MG1655_t0_r[1,2]) and 11 of the remaining 12 experiments were from three separate, entire time series of experiments [See Faith et al., 2007 experimental series ccdB_chelator_MG1063_t[0,30,60,120]_r1, ccdB_BW25113_t[0,30,60,120,180]_r1, ccdB_BW25113recA_t[0,30,60,120,180]_r1; ccdB_MG1063_t120_r1 is the remaining (12th) experiment]. None of these cases had a_ij_ values near 0.5*.

Figure 2A shows the raw expression data (histogram) and an overlaid Gaussian mixture distribution from the MultiMM method for *araB*. The remaining three figures (Figures 2B–D) graph the posterior probability that *araB* is active in experiment *j* (*a*_*ij*_) vs. the log expression value (ϵ_*ij*_). The rank based method (Figure 2B) yields uncertain calls for many of the expression values (many *a*_*ij*_ values near 0.5) for values which are clearly inactive based on the histogram. The UniMM (Figure 2C) and MultiMM (Figure 2D) approaches yield results that directly correspond to the raw expression values shown in the top left histogram. Notably, the MultiMM improves on the call certainty of the UniMM: it takes one somewhat uncertain call from the UniMM approach (*a*_*ij*_ = 0.35) and makes that call more certain by leveraging observations about the experiment from other genes in the operon (where the other genes in the operon are clearly in the inactive cluster; details not shown).

Figure 3A shows the raw expression data (histogram) and an overlaid Gaussian mixture distribution from the MultiMM method for *araA* (Note: a histogram for *araB* is provided in Figure 2A). The remaining three figures (Figures 3B–D) graph the observed log-expression values (ϵ_*ij*_) for *araA* vs. *araB*. *araA* and *araB* are contained within the same operon, a three gene operon that also includes *araD* (not shown). The rank based method (Figure 3B) yields uncertain and inconsistent calls for many of the expression values which appear to be clearly in the inactive category based on the apparent clustering. The UniMM (Figure 3C) and MultiMM (Figure 3D) approaches both show much better performance. Notably, the MultiMM approach eliminates one inconsistent call by leveraging observations about this experiment (the third gene in the operon (*araD*), is clearly in the inactive cluster; details not shown).

#### Cysteine synthase operon

**Figure 4:**
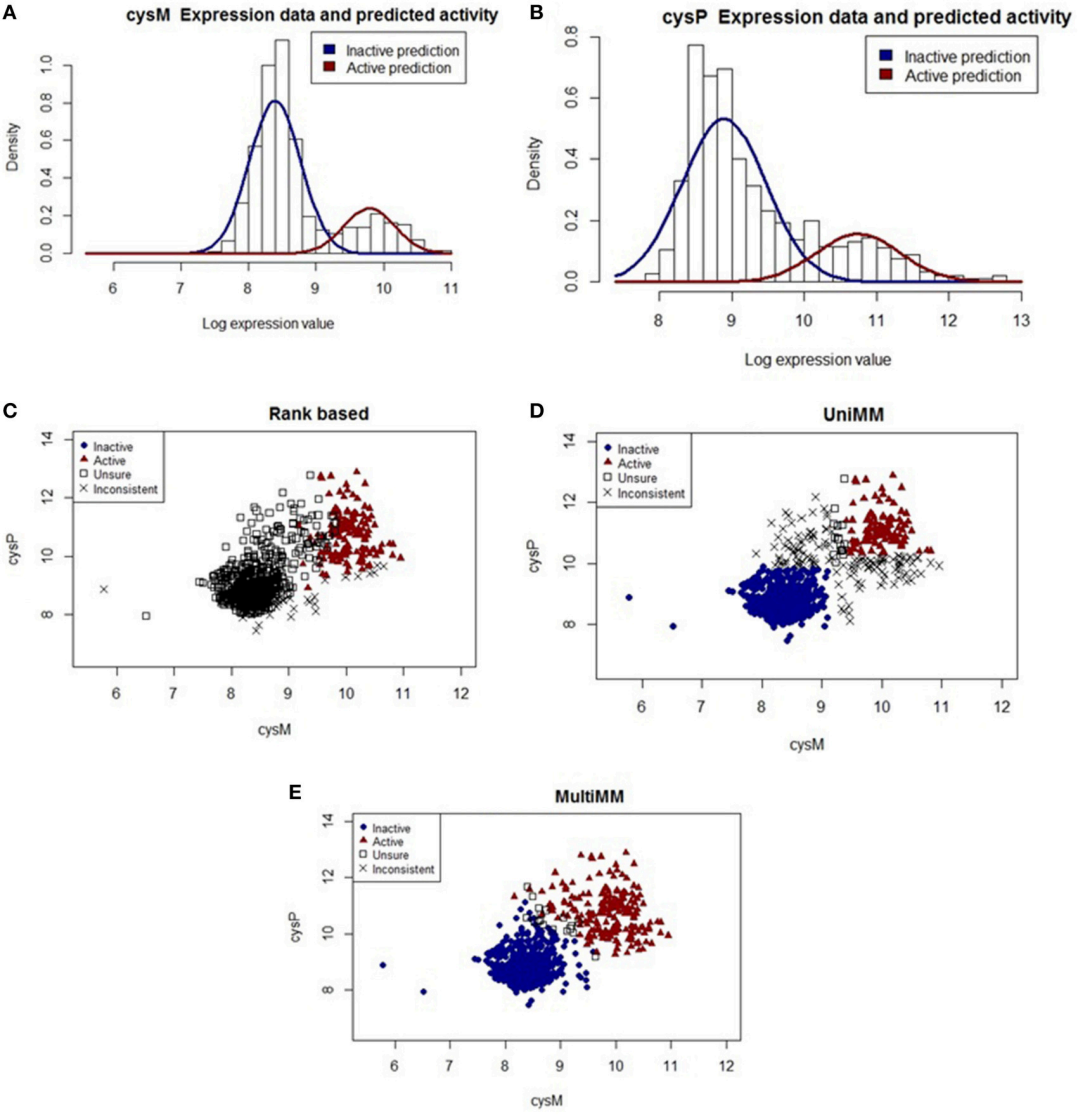
**Performance of different activity state inference methods on gene pair ***cysM*** and ***cysP*****. **(A,B)** show raw expression data with overlaid Gaussian mixture distributions from the MultiMM method for *cysM* and *cysP*, respectively. **(C–E)** graph the observed expression values for these two genes, indicating how consistent the expression values are with three different methods of estimating gene activity states. Blue dots represent experiments for which the approach is very sure the gene pair is inactive (*a*_*ij*_ < 0.2) and red triangles represent experiments for which the approach is very sure the gene pair is active (*a*_*ij*_ > 0.8). Open squares represent unsure (0.2 < *a*_*ij*_ < 0.8) calls for both genes, and black “X's” represent situations where either *a*_*ij*_ < 0.2 for one gene and *a*_*ij*_ > 0.2 for the other, or *a*_*ij*_ < 0.8 for one gene and *a*_*ij*_ > 0.8 for the other. The MultiMM method, by leveraging information from all genes in the operon, is able to provide the most consistent calls of the three methods.

The cysteine synthase operon consists of five genes (*cysM, cysA, cysW, cyst*, and *cysP*) which encode proteins associated with cysteine biosynthesis. Figure 4 illustrates how the *MultiMM* performs better than the *UniMM* and *RB* approaches for genes in this operon.

Figure 4A shows the raw expression data (histogram) and an overlaid Gaussian mixture distribution from the MultiMM method for *cysM*, with a comparable figure for *cysP* (Figure 4B). Note that *cysM* and *cysP* are located within the same operon, which also contains three other genes (*cysA, cysW*, and *cysT*; not shown). It is also important to note that, while the Gaussian mixtures fit the data well, there is less separation in the clusters than is present in the *araA/araB* example (see Figures 2, 3). Furthermore, note that the threshold for active-inactive appears to be at approximately 9 for *cysM* (Figure 4A) but is higher (~10.5) for *cysP* (Figure 4B). Figures 4C–E graph the observed log-expression values (ϵ_*ij*_) for *cysM* vs. *cysP*. The rank based method (Figure 4C) yields uncertain and inconsistent calls for many of the expression values which appear to be clearly in the inactive category based on the apparent clustering. The UniMM (Figure 4D) and MultiMM (Figure 4E) approaches both show much better performance, though the UniMM approach yields numerous inconsistent calls. Notably, the MultiMM approach eliminates all inconsistent calls by leveraging observations about each experiment from the other genes in the operon (details not shown).

The relatively “high” expression values observed when genes in the cysteine synthase operon are in the inactive state lead to poor performance of the rank-based method. Furthermore, the increased within gene-state variability yields more uncertain calls from the *UniMM* method which leads to a large number of inconsistent gene calls for genes in this operon. The *MultiMM* method leverages operonal structure to consistently infer gene states for all genes in the operon. In the majority of cases when the experiment was performed in the presence of yeast extract, a rich media, the *MultiMM* method identified the operon as inactive (91.3%; 625/684); presumably, in these conditions, cysteine would not need to be synthesized by the cell. Relatedly, when the experiment was not performed in the presence of yeast extract, the cysteine synthase operon was typically identified as active by the *MultiMM* approach (68.6%; 153/223). The *UniMM* approach yielded similar results, but other methods showed much weaker association between the yeast extract media and cysteine synthase activity.

#### Overall patterns of gene activity

Overall, the *MultiMM* method yielded 3538 genes which were determined to be in both active and inactive states at least once in the set of 907 experiments, with 791 genes that did not show evidence of changing states in this set of experiments. There was large variation among the 3538 genes in values of ${\hat{\mu }}_{0}$ (Estimated mean of the inactive state expression values; Min = 3.63, Q1 = 7.43, Median = 8.08, Q3 = 8.65, Max = 13.09), ${\hat{\mu }}_{1}$ (Estimated mean of the active state expression values; Min = 4.85, Q1 = 8.32, Median = 9.22, Q3 = 10.14, Max = 14.46), Σ(Estimated standard deviation of expression values within each state; Min = 0.19, Q1 = 0.35, Median = 0.45, Q3 = 0.58, Max = 1.91), and $\hat{\pi }$ (Estimated proportion of times the gene is active across all experiments in the set; Min = 0.01, Q1 = 0.10, Median = 0.26, Q3 = 0.82, Max = 0.99).

Among 684 experiments done in the presence of yeast extract (a rich media), the overall (across all genes and experiments) average *a*_*ij*_ value was 0.418, compared to 0.428 among the 223 experiments performed without yeast extract (*p* = 0.002). To better understand which aspects of the metabolic network may be accounting for this difference, we used a gene set analysis approach to test for potential differences in average activity level for pathway components (see methods) between the different sets of experiments. Supplemental Table 1 provides the full list of 161 pathway components. Notably, 29 of the top 40 most positively differentially expressed pathway components (more activity when yeast extract was absent than when it was present) involve synthesis of metabolic components (*p* < 0.002 in all cases) compared to only six of the top 35 most negatively differentially expressed pathway components (more activity when yeast extract was present; *p* < 0.002 in all cases).

Furthermore, Supplemental Table 1 includes a column indicating whether or not a pathway component is related to amino acid biosynthesis according to the SEED (DeJongh et al., 2007). Eighteen of the 19 amino acid biosynthesis pathway components are in the top 44 most positively differentially expressed pathway components, as would be expected given that these pathway components are typically not needed in the presence of yeast extract.

#### Evaluation of gene activity state estimates and experimentally measured reaction fluxes

Lastly, we evaluated different methods of estimating gene activity states vs. experimentally measured reaction fluxes. Table 8 summarizes the results of these analyses. The *MultiMM* method yielded the strongest correlation between experimental measured fluxes and gene activity state estimates (0.354; 95 CI: 0.303 to 0.406; *p* < 0.001). A multiple regression model predicting flux measurements by both *MultiMM* and raw gene expression values found that while *MultiMM* is significantly associated with flux values (Std Beta = 0.344; 95% CI: 0.280, 0.480; *p* < 0.001), raw expression values are not unique predictors of flux values (Std. Beta = 0.018; 95% CI: -0.046, 0.082; *p*>0.05), suggesting that the *MultiMM* method has sufficiently captured the aspects of expression data which associate with flux. We note (see Table 8 for details), that this is also true of the TT method, but not the MT and RB methods. Importantly, however, in models predicting fluxes using both the *MultiMM* method and other gene activity estimates, the *MultiMM* method explains substantially more variation in flux values than other methods [between 0.29 and 0.38 vs. −0.032 (MT), 0.085 (TT) and 0.093 (RB)]. Because of the high correlation between the *UniMM* and *MultiMM* methods on this dataset (see Table 8, footnote D) we only focused on the *MultiMM* method in the previous paragraph.

**Table 8:** **Association between inferred gene activity states and experimentally measured fluxes**.

**Method**	**Correlation with flux (95% CI)^a^**	**Multiple regression with ϵ**_ij_		**Multiple regression with** ***MultiMM***	
		**Partial correlation of gene activity estimate with flux (95% CI)^b^**	**Partial correlation of expression data with flux (95% CI)^b^**	**Partial correlation of gene activity estimate with flux (95% CI)^c^**	**Partial correlation of *MultiMM* activity estimate with flux (95% CI)^c^**
Raw expression (ϵ_ij_)	0.223 (0.170 to 0.277)^***^	−	−	0.018 (−0.046 to 0.082)	0.344 (0.280 to 0.408)^***^
MT	0.255 (0.202 to 0.308)^***^	0.189 (0.123 to 0.255)^***^	0.111 (0.045 to 0.177)^***^	−0.032 (−0.110 to 0.047)	0.378 (0.300 to 0.457)^***^
TT	0.311 (0.25 to 0.364)^***^	0.296 (0.225 to 0.367)^***^	0.023 (−0.045 to 0.094)	0.085 (0.002 to 0.168)^*^	0.287 (0.204 to 0.370)^***^
RB	0.305 (0.253 to 0.358)^***^	0.417 (0.318 to 0.517)^***^	−0.132 (−0.231 to −0.032)^**^	0.093 (0.017 to 0.170)^*^	0.285 (0.208 to 0.361)^***^
UniMM	0.351 (0.300 to 0.403)^***^	0.336 (0.273 to 0.399)^***^	0.026 (−0.037 to 0.089)	−^d^	−^d^
MultiMM	0.354 (0.303 to 0.406)^***^	0.344 (0.280 to 0.408)^***^	0.018 (−0.046 to 0.082)	−	−

*** p < 0.001;

** p < 0.01;

* *p < 0.05*.

a *These standardized beta coefficients (i.e., correlations) result from predicting flux values by either the raw expression data or gene activity state estimates*.

b *These standardized beta coefficients (i.e., partial correlations) result from predicting flux values by one of the gene activity estimates and the raw expression data. When the partial correlation for expression data is significant it suggests that the corresponding gene activity estimating method is not sufficiently capturing the variation in expression data that explains changes in flux*.

c *These standardized beta coefficients (i.e., partial correlations) result from predicting flux values by one of the gene activity estimates and the MultiMM approach. The partial correlations for the MultiMM method are always much larger and more significant compared to other gene activity approaches, suggesting that the MultiMM method is explaining significantly more variation in flux values than other approaches*.

d *The correlation between UniMM and MultiMM activity estimates on this dataset is 0.998 (essentially equivalent) making linear models containing both UniMM and MultiMM activity estimates lack robustness*.

## Discussion

We have presented a Bayesian framework for the classification of microbial gene activity states (active or inactive), based on a compendium of genome-wide gene expression data. Our approach first uses the Bayesian Information Criterion to identify genes that likely have a mixture of both active and inactive states present in the data. A Gibbs sampler is then used to provide estimates of the posterior probability that a gene is active in each condition, based on a Gaussian normal mixture model. Our approach addresses four key limitations of existing approaches for classifying gene activity states: (a) different activity thresholds for different genes (Figure 4A vs. Figure 4B), (b) different proportions of gene activity between different experiments (*Results, Overall patterns of gene activity*), (c) benefits of leveraging a priori evidence of co-regulation (Figures 4C–E) and (d) benefits of quantified statistical uncertainty (e.g., Figure 1, among others). Specific figures and tables in the manuscript provide visual intuition about how the proposed method addresses these limitations.

By addressing these limitations, the Mixture Model approaches (*MultiMM* and *UniMM*) show less deviation from true gene activity states on simulated data, more consistency with metabolic model flux predictions and operon structure on real data, and stronger association with experimentally measured fluxes, with *MultiMM* doing best. Results from model fitting on the real data by the mixture model approaches showed great variance in the overall means, standard deviations and mixing proportions suggesting empirically that the limitations stated above are, in fact, realistic concerns for existing approaches which can be addressed by these mixture model approaches. Furthermore, association between inferred gene activity states using the *MultiMM* approach yielded stronger correlations with observed flux data, while other methods *(MT, TT*, and *RB*, as well as the raw expression data itself) left substantial variation in flux values as unexplained. Finally, pathway components associated with synthesis activities were significantly more expressed in the absence of a rich media condition (yeast extract) than in the presence of a rich medium as expected.

The improved performance of *MultiMM* over *UniMM* highlights the utility of incorporating genome-based operon predictions in activity state estimation. The Bayesian approach we have developed acts as a general framework for future innovation via the inclusion of other –omics data sources. For example, transcriptional regulatory networks (TRNs) can be actively incorporated into the analysis pipeline by expanding the *MultiMM* approach to utilize regulons in addition to operons. However, full integration of TRN information will require explicitly incorporating TRN uncertainty into the Bayesian framework. For example, due to TRN uncertainty we might be only 90% sure that two genes are co-regulated, in contrast to our current approach, which requires gene sets (operons) to be defined explicitly and with 100% certainty. Furthermore, this same uncertainty approach can likely be applied to operons when, for example, an operon comprises several transcription units. We believe the Bayesian framework provided here provides a flexible platform for this future innovation, in order to continue to reduce overall deviation of activity state estimates from true gene activity states. A similar model is also being explored by our group to incorporate (a) flux profiles, (b) functional information, (c) cross-organism gene homology and other sources of genetic information which, when incorporated into the activity state estimates, may further improve their accuracy and precision.

Downstream applications of improved gene activity state measurements are numerous, though we focus our discussion here mainly on metabolic modeling. In particular, our improved gene activity state measurements will allow us to incorporate gene activity information into subsequent metabolic models simulations through statistically informed penalties, rather than arbitrary or loose penalties, which often serve to down-weight gene expression data to the point where it has little to no real impact on downstream modeling results (e.g., Chandrasekaran and Price, 2010). We are currently exploring metabolic modeling advances that incorporate *a*_*ij*_'s.

Some limitations of our approach and our analysis here are worth noting. Gene activity state estimates likely improve as the number and diversity of experimental conditions increases, though we saw promising results even in a small follow-up study of expression data in 29 conditions. Given the dramatically reduced price of RNA-sequencing, continued rapid growth in the size and diversity of expression data is expected to make this limitation less of an issue. Our analysis here focuses on *E. coli*, though an important area of future work involves application of our approaches to a variety of other microbes, both to demonstrate transferability of the approach, but also in order to explore methods of improving activity state inference by leveraging information from multiple organisms simultaneously (e.g., gene homology, regulatory and metabolic homology, etc.). Further work is needed to evaluate the performance of these methods on RNA-seq data, though we anticipate the performance should be similar after standard normalization and transformation procedures are applied to the data. Finally, numerous additional validation studies are possible (e.g., an expanded comparison to observed flux data) and should be considered in future work. An initial small-scale evaluation presented here showed promising initial results.

## Conclusions

We have developed a flexible Bayesian framework for the estimation of gene activity states from compendia of microbial gene expression data. Our approach provides improved consistency with true gene activity states compared to existing approaches on a large compendia of *E coli* expression data. Future work is needed to evaluate the performance of the method on other organisms and to expand the Bayesian model presented to incorporate other –omics data sources.

## Author contributions

NT, MD, and AB contributed to the initial design of the study and motivating research questions. NT, CD, BG, KC, KK, RL, CV, JC, EH, YA, and KF designed the method, developed evaluative metrics, simulated data and implemented the methods. AA, MC, AB, and MD contributed to the evaluation of data, implementation and running of the metabolic models and understanding and parsing of media conditions. NT, CD, BG, and KC drafted initial versions of the manuscript. All authors saw, edited and approved of the final manuscript.

### Conflict of interest statement

The authors declare that the research was conducted in the absence of any commercial or financial relationships that could be construed as a potential conflict of interest. The reviewer JZ and handling Editor declared their shared affiliation, and the handling Editor states that the process nevertheless met the standards of a fair and objective review.

## Footnotes

^1^GIMME software page [http://csbl.bitbucket.org/tiger/doc/tiger/tie/gimme.html]

^2^mclust: Normal mixutre modeling for model-based clustering, classification and density estimation [https://cran.r-project.org/web/packages/mclust/index.html]

^3^Many Microbes Database. [http://m3d.mssm.edu]

^4^Affymetrix. [http://www.affymetrix.com]

## References

[B1] Abel S. Bucher T. Nicollier M. Hug I. Kaever V. Abel zur Wiesch P.. (2013). Bi-modal distribution of the second messenger c-di-GMP controls cell fate and asymmetry during the caulobacter cell cycle. PLoS Genet. 9:e1003744. 10.1371/journal.pgen.1003744 24039597 PMC3764195

[B2] Aziz R. K. Bartels D. Best A. A. DeJongh M. Disz T. Edwards R. A.. (2008). The RAST Server: rapid annotations using subsystems technology. BMC Genomics 9:75. 10.1186/1471-2164-9-75 18261238 PMC2265698

[B3] Becker S. A. Palsson B. O. (2008). Context-specific metabolic networks are consistent with experiments. PLoS Comput. Biol. 4:e1000082. 10.1371/journal.pcbi.1000082 18483554 PMC2366062

[B4] Bordbar A. Monk J. M. King Z. A. Palsson B. O. (2014). Constraint-based models predict metabolic and associated cellular functions. Nat. Rev. Genet. 15, 107–120. 10.1038/nrg3643 24430943

[B5] Chalancon G. Ravarani C. N. J. Balaji S. Martinez-Arias A. Aravind L. Jothi R.. (2012). Interplay between gene expression noise and regulatory network architecture. Trends Genet. 28, 221–232. 10.1016/j.tig.2012.01.006 22365642 PMC3340541

[B6] Chandrasekaran S. Price N. D. (2010). Probabilistic integrative modeling of genome-scale metabolic and regulatory networks in Escherichia coli and *Mycobacterium tuberculosis*. Proc. Natl. Acad. Sci. U.S.A. 107, 17845–17850. 10.1073/pnas.1005139107 20876091 PMC2955152

[B7] Chubukov V. Gerosa L. Kochanowski K. Sauer U. (2014). Coordination of microbial metabolism. Nat. Rev. Microbiol. 12, 327–340. 10.1038/nrmicro3238 24658329

[B8] Colijn C. Brandes A. Zucker J. Lun D. S. Weiner B. Farhat M. R.. (2009). Interpreting expression data with metabolic flux models: predicting *Mycobacterium tuberculosis* mycolic acid production. PLoS Comput. Biol. 5:e1000489. 10.1371/journal.pcbi.1000489 19714220 PMC2726785

[B9] DeJongh M. Formsma K. Boillot P. Gould J. Rycenga M. Best A. (2007). Toward the automated generation of genome-scale metabolic networks in the SEED. BMC Bioinformatics 8:139. 10.1186/1471-2105-8-139 17462086 PMC1868769

[B10] Faith J. J. Driscoll M. E. Fusaro V. A. Cosgrove E. J. Hayete B. Juhn F. S.. (2008). Many microbe microarrays database: uniformly normalized affymetrix compendia with structured experimental metadata. Nucleic Acids Res. 36(Database issue), D866–D870. 10.1093/nar/gkm815 17932051 PMC2238822

[B11] Faith J. J. Hayete B. Thaden J. T. Mogno I. Wierzbowski J. Cottarel G.. (2007). Large-scale mapping and validation of *Escherichia coli* transcriptional regulation from a compendium of expression profiles. PLoS Biol. 5:e8. 10.1371/journal.pbio.0050008 17214507 PMC1764438

[B12] Fang X. Wallqvist A. Reifman J. (2012). Modeling phenotypic metabolic adaptations of *Mycobacterium tuberculosis* H37Rv under Hypoxia. PLoS Comput. Biol. 8:e1002688. 10.1371/journal.pcbi.1002688 23028286 PMC3441462

[B13] Ferrell J. E. (2002). Self-perpetuating states in signal transduction: positive feedback, double-negative feedback and bistability. Curr. Opin. Cell Biol. 14, 140–148. 10.1016/S0955-0674(02)00314-9 11891111

[B14] Fraley C. Raftery A. E. (2007). Bayesian regularization for normal mixture estimation and model-based clustering. J. Classif. 24, 155–181. 10.1007/s00357-007-0004-5

[B15] Gallo C. A. Cecchini R. L. Carballido J. A. Micheletto S. Ponzoni I. (2015). Discretization of gene expression data revised. Brief. Bioinform. 1–13 10.1093/bib/bbv074. [Epub ahead of print]. 26438418

[B16] Gamba P. Jonker M. J. Hamoen L. W. (2015). A novel feedback loop that controls bimodal expression of genetic competence. PLoS Genet. 11:e1005047. 10.1371/journal.pgen.1005047 26110430 PMC4482431

[B17] Henry C. S. DeJongh M. Best A. A. Frybarger P. M. Linsay B. Stevens R. L. (2010). High-throughput generation, optimization and analysis of genome-scale metabolic models. Nat. Biotechnol. 28, 977–982. 10.1038/nbt.1672 20802497

[B18] Irizarry R. A. Bolstad B. Collin F. Cope L. Hobbs B. Speed T. (2003). Summaries of affymetrix genechip probe level data. Nucleic Acids Res. 31:e15. 10.1093/nar/gng015 12582260 PMC150247

[B19] Ishii N. Nakahigashi K. Baba T. Robert M. Soga T. Kanai A.. (2007). Multiple high-throughput analyses monitor the response of *E. coli* to perturbations. Science 316, 593–597. 10.1126/science.1132067 17379776

[B20] Jensen P. A. Papin J. A. (2011). Functional integration of a metabolic network model and expression data without arbitrary thresholding. Bioinformatics 27, 541–547. 10.1093/bioinformatics/btq702 21172910 PMC6276961

[B21] Jensen P. A. Lutz K. A. Papin J. A. (2011). TIGER: Toolbox for integrating genome-scale metabolic models, expression data, and transcriptional regulatory networks. BMC Syst. Biol. 5:147. 10.1186/1752-0509-5-147 21943338 PMC3224351

[B22] Jerby L. Ruppin E. (2012). Predicting drug targets and biomarkers of cancer via genome-scale metabolic modeling. Clin. Cancer Res. 18, 5572–5584. 10.1158/1078-0432.CCR-12-1856 23071359

[B23] Kim J. Reed J. L. (2012). RELATCH: relative optimality in metabolic networks explains robust metabolic and regulatory responses to perturbations. Genome Biol. 13:R78. 10.1186/gb-2012-13-9-r78 23013597 PMC3506949

[B24] Lee D. Smallbone K. Dunn W. B. Murabito E. Winder C. L. Kell D. B.. (2012). Improving metabolic flux predictions using absolute gene expression data. BMC Syst. Biol. 6:73. 10.1186/1752-0509-6-73 22713172 PMC3477026

[B25] Lewis N. E. Nagarajan H. Palsson B. O. (2012). Constraining the metabolic genotype-phenotype relationship using a phylogeny of *in silico* methods. Nat. Rev. Microbiol. 10, 291–305. 10.1038/nrmicro2737 22367118 PMC3536058

[B26] Losick R. Desplan C. (2008). Stochasticity and cell fate. Science 320, 65–68. 10.1126/science.1147888 18388284 PMC2605794

[B27] Machado D. Herrgård M. (2014). Systematic evaluation of methods for integration of transcriptomic data into constraint-based models of metabolism. PLoS Comput. Biol. 10:e1003580. 10.1371/journal.pcbi.1003580 24762745 PMC3998872

[B28] Mahadevan R. Schilling C. (2003). The effects of alternate optimal solutions in constraint-based genome-scale metabolic models. Metab. Eng. 5, 264–276. 10.1016/j.ymben.2003.09.002 14642354

[B29] Monk J. Nogales J. Palsson B. O. (2014). Optimizing genome-scale network reconstructions. Nat. Biotechnol. 32, 447–452. 10.1038/nbt.2870 24811519

[B30] Morfopoulou S. Plagnol V. (2015). Bayesian mixture analysis for metagenomic community profiling. Bioinformatics 31, 2930–2938. 10.1093/bioinformatics/btv317 26002885 PMC4565032

[B31] Moxley J. F. Jewett M. C. Antoniewicz M. R. Villas-Boas S. G. Alper H. Wheeler R. T.. (2009). Linking high-resolution metabolic flux phenotypes and transcriptional regulation in yeast modulated by the global regulator Gcn4p. Proc. Natl. Acad. Sci. U.S.A. 106, 6477–6482. 10.1073/pnas.0811091106 19346491 PMC2672541

[B32] Murphy K. P. (2007). Conjugate Bayesian Analysis of the Gaussian Distribution. Technical Report, University of British Columbia Available online at: https://www.cs.ubc.ca/~murphyk/Papers/bayesGauss.pdf

[B33] Navid A. Almaas E. (2012). Genome-level transcription data of Yersinia pestis analyzed with a new metabolic constraint-based approach. BMC Syst. Biol. 6:150. 10.1186/1752-0509-6-150 23216785 PMC3572438

[B34] Ohtaki M. Otani K. Hiyama K. Kamei N. Satoh K. Hiyama E. (2010). A robust method for estimating gene expression states using Affymetrix microarray probe level data. BMC Bioinformatics 11:183. 10.1186/1471-2105-11-183 20380745 PMC2873532

[B35] Orth J. D. Conrad T. Na J. Lerman J. Nam H. Feist A.. (2011). A comprehensive genome-scale reconstruction of *Escherichia coli* metabolism - 2011. Mol. Syst. Biol. 11:535. 10.1038/msb.2011.65 21988831 PMC3261703

[B36] Pfau T. Christian N. Ebenhoh O. (2011). Systems approaches to modelling pathways and networks. Brief. Funct. Genomics 10, 266–279. 10.1093/bfgp/elr022 21903724

[B37] Powers S. De Jongh M. Best A. A. Tintle N. L. (2015). Cautions about the reliability of pairwise gene correlations based on expression data. Front. Microbiol. 6:650. 10.3389/fmicb.2015.00650 26167162 PMC4481165

[B38] Price M. N. Huang K. H. Alm E. J. Arkin A. P. (2005). A novel method for accurate operon predictions in all sequenced prokaryotes. Nucleic Acids Res. 33, 880–892. 10.1093/nar/gki232 15701760 PMC549399

[B39] Raftery A. (1995). Bayesian model selection in social research. Soc. Methods 25, 111–163. 10.2307/271063

[B40] Rezola A. Pey J. Tobalina L. Rubio A. Beasley J. E. Planes F. J. (2014). Advances in network-based metabolic pathway analysis and gene expression data integration. Brief. Bioinform. 16:bbu009. 10.1093/bib/bbu009 24626528

[B41] Rubin D. B. (1987). Multiple Imputation for Nonresponse in Surveys. 1st Edn. New York, NY: John Wiley and Sons 10.1002/9780470316696

[B42] Schleif R. (2010). AraC protein, regulation of the l-arabinose operon in *Escherichia coli*, and the light switch mechanism of AraC action. FEMS Microbiol. Rev. 34, 779–796. 10.1111/j.1574-6976.2010.00226.x 20491933

[B43] Schmidt B. J. Ebrahim A. Metz T. O. Adkins J. N. Palsson B. Ø. Hyduke D. R. (2013). GIM3E: condition-specific models of cellular metabolism developed from metabolomics and expression data. Bioinformatics 29, 2900–2908. 10.1093/bioinformatics/btt493 23975765 PMC3810847

[B44] Shlomi T. Cabili M. N. Herrgård M. J. Palsson B. Ø. Ruppin E. (2008). Network-based prediction of human tissue-specific metabolism. Nat. Biotechnol. 26, 1003–1010. 10.1038/nbt.1487 18711341

[B45] Tintle N. L. Best A. A. DeJongh M. Van Bruggen D. Heffron F. Porwollik S.. (2008). Gene set analyses for interpreting microarray experiments on prokaryotic organisms. BMC Bioinformatics 9:469. 10.1186/1471-2105-9-469 18986519 PMC2587482

[B46] Tintle N. Sitarik A. Boerema B. Young K. Best A. De Jongh M. (2012). Evaluating the consistency of gene sets used in the analysis of bacterial gene expression data. BMC Bioinformatics 13:193. 10.1186/1471-2105-13-193 22873695 PMC3462729

[B47] Van Berlo R. J. P. De Ridder D. Daran J. Daran-lapujade P. A. S. Teusink B. Reinders M. J. T. (2011). Predicting metabolic fluxes using gene expression differences as constraints. IEEE/ACM Trans. Comput. Biol. Bioinform. 8, 206–216. 10.1109/tcbb.2009.55 21071808

